# Psychiatric patients turnaround times in the emergency department

**DOI:** 10.1186/1745-0179-1-27

**Published:** 2005-12-13

**Authors:** Stefan Kropp, Christoph Andreis, Bert te Wildt, Udo Reulbach, Martin Ohlmeier, Irina Auffarth, Marc Ziegenbein

**Affiliations:** 1 Department of Psychiatry, Psychotherapy and Psychosomatics, Landesklinik Teupitz, 15755 Teupitz, Germany; 2 Department of Clinical Psychiatry and Psychotherapy, Hannover Medical School, 30623 Hannover, Germany; 3 Departement of Psychiatry and Psychotherapy, University Erlangen-Nuremberg, Germany; 4 Department of Social Psychiatry and Psychotherapy, Hannover Medical School, 30623 Hannover, Germany

## Abstract

**Background:**

To analyze the turnaround times of psychiatric patients within the Emergency Department (ED) from registration to discharge or hospitalization in a University Hospital in 2002.

**Methods:**

Data from a one-year period of psychiatric admissions to the emergency service at a University Hospital were monitored and analyzed focused on turnaround times within the ED. Information on patients variables such as age, sex, diagnosis, consultations and diagnostic procedures were extracted from the patients' charts.

**Results:**

From 34.058 patients seen in the ED in 2002, 2632 patients were examined by psychiatrists on duty. Mean turnaround time in the ED was 123 (SD 97) minutes (median 95). Patients to be hospitalized on a psychiatric ward stayed shorter within the ED, patients who later were admitted to another faculty, were treated longer in the ED. Patients with cognitive or substance related disorders stayed longer in the ED than patients with other psychiatric diagnoses. The number of diagnostic procedures and consultations increased the treatment time significantly.

**Conclusion:**

As the number of patients within the examined ED increases every year, the relevant variables responsible for longer or complicated treatments were assessed in order to appropriately change routine procedures without loss of medical standards. Using this basic data, comparisons with the following years and other hospitals will help to define where the benchmark of turnaround times for psychiatric emergency services might be.

## Background

Turnaround time is an important parameter that strongly influences patients and staff satisfaction in the emergency department and there are early reports considering this important issue [1]. In the department examined it is defined as the time from patient arrival to either discharge or hospitalization. The measurement of turnaround time is an helpful variable of efficacy which is feasible in most emergency departments. Measuring turnaround times may have two major goals: improving the medical care delivered within reasonable time and taking care of for patients' satisfaction [2-5]. Adhering to a comprehensive philosophy of quality management today is vital for hospitals to compete with other hospitals and healthcare providers. The quality of health care has many different components and is difficult to define. One fundamental principle of quality management is that quality can best be improved if it is measured and compared [6]. Within the emergency department, the measurement of psychiatric service quality with different quality indicators might be useful. As waiting and turnaround times might be crucial to the outcome of a medical disease and the reduction of waiting times will positively influence patients perceptions of a hospital and its services, the purpose of this study is to examine the turnaround times of psychiatric patients according to general and diagnose-related variables as a basis of one possible quality indicator.

## Methods

For the period of one year, all psychiatric emergency consultations (n = 2632) within the ED of the Hanover Medical School in Germany were retrospectively monitored, including the time of registration and discharge from ED. We assessed general patients variables and diagnostic procedures in the ED, which might have an impact on the turnaround times. Each procedure such as physical examination, laboratory tests, ECG, X-ray or cranial CT-scan counted as one item. When patients refused physical examination and no other procedures were done, the number of items was zero. All statistical analyses were performed with SPSS™ 12. Besides descriptive statistics, nonparametric methods such as Pearson's Chi Quare test, Mann-Whitney-U-test, Kruskal-Wallis-test and correlation analysis by Spearman were performed. All tests were two-sided. The significance level was set at α 00.05 or less.

## Results

**Figure 1:**
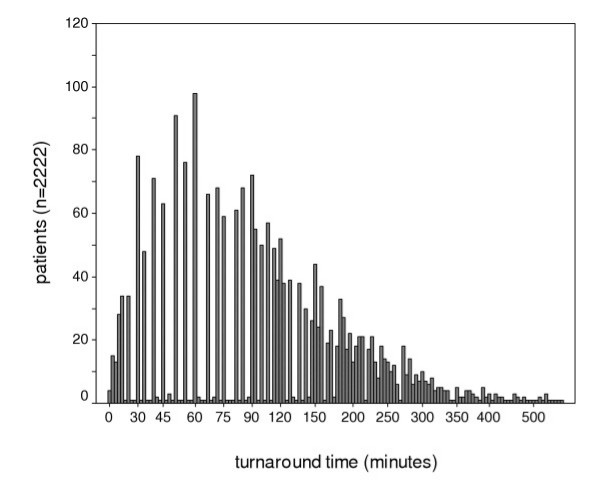
**Turnaround times**. Turnaround times of the general population of 2222 psychiatric patients within the emergency department in the year 2002.

2632 patients were assessed, 48.4% were female. The mean age was 43.5 (SD 16.0) years. Female patients were significantly older than male patients (Mann-Whitney-U, Z=-3.4, p = 0.001). 567 patients were secondly referred to a psychiatrist by faculties for consultation (e.g. internal medicine, neurology, surgery). 945 patients were admitted to psychiatric wards and 104 patients to non psychiatric wards. 107 patients refused hospitalization. Substance-related problems (ICD-10 F1X, 672 patients) and psychotic disorders including schizophrenia (ICD-10 F2X, 391 patients) were the most common diagnoses, followed by somatoform, anxiety and neurotic disorders (ICD-10 F4X, 332 patients). 104 patients were involuntary admitted acording to PsychKG (German law concerning psychiatric practice). In 2222 patients, the time from door to release or to hospitalisation was determined. The mean time of stay in ED was 123 (SD 97) minutes, median 95 minutes. 77.9% of all psychiatric patients were released or hospitalized within 3 hours, 88.3% within 4 hours (Fig. 1).

To acknowledge possible influence factors, the first analysis was done taking into account working shifts within the staff of the ED. During the day shift, 26.8% of the patients stayed for more than 3 hours in the ED, during the night shift only 17.9% had to stay that long (Chi-Square = 19.8, df = 2, p < 0.001). By dividing the day into six four hour periods, significant differences between the categorized length of stay could be detected (Chi-Square = 55.6, df = 10, p < 0.001). Between midnight and 4 h, the percentage of patients staying longer than three hours dropped to 7,5%. We found no difference between working days or weekends (Chi-Quare = 0.03, df = 2, p = 0.986). Patients who were hospitalized stayed longer than the discharged patients (Mann-Whitney-U, Z=-3.2, p = 0.002). Patients admitted via the ED to a psychiatric ward stayed shorter in the ED than patients who were admitted to another faculty, for example 14.2% of the later admitted psychiatric patients stayed 3 or more hours in the ED. But 25.9% of the patients admitted to other faculties (Chi-Square = 60.4, df = 2, p = <0.001). Patients who belonged to the urban catchment area were discharged significantly more often within an hour than patients from outside the catchment area (28.0% vs. 21.0%; Chi-Square = 13.3, p = 0.001).

The established diagnosis had a significant impact on turnaround times (Chi-Square = 154.3, df = 14, p < 0.001): patients with dementia or cognitive disorders often stayed longer than three hours in the ED (51.3%) and they were less likely to stay only one hour (11.5%) or even 1 to 3 hours in the ED (37.2%). Patients with substance-related disorders were treated less likely only up to one hour within the emergency department (16.8%). The group of patients with schizophrenia or personality disorders often stayed only up to one hour in the ED (schizophrenia 42.8%; personality disorders 32.8%). The groups of patients with anxiety, mood or adjustment disorders, or other psychiatric disorders did not reveal relevant differences from the mean time of all patients within the ED.

**Figure 2:**
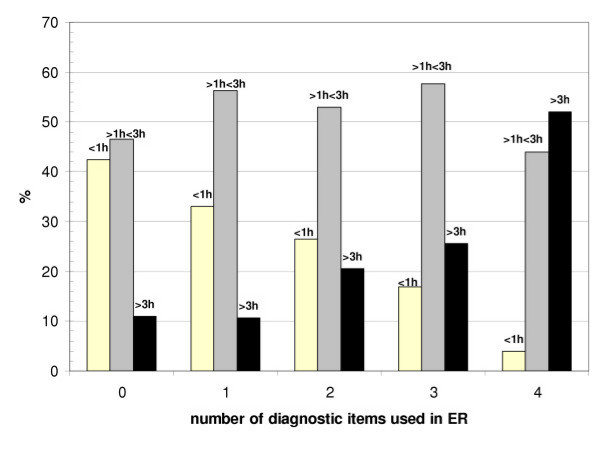
**Diagnostic procedures and time**. Turnaround times in percent of time intervals according to the number of diagnostic procedures provided to the population of psychiatric patients (0 – 4 procedures).

**Figure 3:**
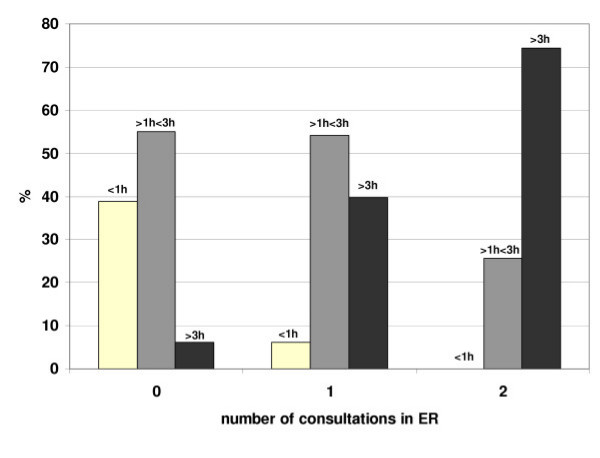
**Consultations**. Turnaround times in percent of time intervals according to the number of consultations given to psychiatric patients by other facultys (none up to 2 consultations by different faculties).

As shown in Fig. 2, with more diagnostic procedures performed, the time patients had to stay within the ED increased significantly (Spearman's rho = 0.132, p = <0.001). When patients were referred for consultations to other faculties (i.e. neurology, internal medicine, surgery) the time within the ED also increased according to the number of consultations as shown in Fig. 3 (Kruskal-Wallis-H, Chi-Square = 489.9, df = 2, p = <0.001).

## Discussion

There is little information of turnaround times within psychiatric services in general EDs. In one sample from Bern, Switzerland [7], the mean length of stay was 77 minutes, but treatment seemed to be organized very differently and the number of psychiatric patients per year was also very different, so a real comparison can not be drawn to this data. But data on turnaround times within general EDs is also hard to obtain. It is of general knowledge, that shorter turnaround times are occurring in rural hospitals and the longest times occur in major academic centers with more than 400 beds. The average of general turnaround time for major teaching hospitals was 204 minutes (median 210 minutes) [8]. In another major academic hospital, the average time for later admitted patients was 330, for discharged patients 123 minutes [9]. For all hospitals in the US, the average waiting time is reported to be 5.8 hours in hospitals with overcrowded EDs [10].

In contrast to only examining the waiting time in the ED until the exact moment medical treatment starts, we calculated the turnaround times of the psychiatric service in the general emergency department of a major academic center providing medical emergency services for nearly every medical subdicipline. From our medical understanding of a psychiatric emergency as well as from the patients' perspective the total time spent in ED – including sufficient treatment and discharge or hospitalization – is vital. Therefore, we retrospectively evaluated the complete process concerning different psychiatric diagnoses and other variables. Women were significantly older than men. This circumstance could be explained by the fact that women are more frequently longer socially integrated, which may be related to differential use of mental health services by men and women. The mean turnaround time of 123 minutes seems quite long compared to the data from Switzerland [7]. Compared to the waiting time without treatment and the phenomenon of overcrowded EDs in the US [10,11], it seems rather short. More interestingly, the data concerning different diagnoses and procedures: patients with dementia stay longer than any other group within the ED, which might be related to other medical conditions which also need treatment and assessment. This also seems to be the case with substance related disorders, where patients often need treatment by other faculties. Patients with personality or schizophrenic disorders seem to be treated faster than any other group. Many of these patients were already known and belong to the urban catchment area. Usually they come for crisis intervention and dont need treatment by other faculties. These points could explain the short turnaround times. Often consultations by other faculties and diagnostic procedures are needed to help and treat psychiatric patients adequately, in some cases these additional examinations might be done for legal reasons. In all cases, every consultation and additional examination expands the turnaround time.

As have been recently shown, further research is needed concerning the underlying variables of turnaround times, clinician-based decisions and the quality of care of psychiatric emergency services [12]. The results should be discussed carefully and controversial and are only part of a mosaic of quality dimensions. Patient and staff satisfaction is only one dimension of quality and remain less important than the objective adequacy and accuracy of clinical management according to standard medical criteria [13]. Practioners are at least obligated to provide the most effective care most efficiently. Only in terms of cost-effectiveness we must discuss how to maintain a good standard of care containing costs. Or in the contrary a long turnaround time may be the result of problems in the management of resources and this may influence the health results. For our perspective the health care professional must take into account patient preferences as well as social preferences in assesing and assuring quality [14].

## Conclusion

Although competition between hospitals and health care providers in Europe now starts to be recognized, few data is available on turnaround times of psychiatric services within general emergency departments. As patients and staff satisfaction or dissatisfaction is strongly correlated not only with the medical treatment but also with turnaround times we have chosen this simple measurement in combination with the psychiatric diagnosis and easily obtainable variables to start evaluating the quality of our service to patients. With this first data, comparisons with following years and other hospitals will help to define where the benchmark of turnaround times for psychiatric patients might be.

## Competing interests

The author(s) declare that they have no competing interests.

## Authors' contributions

SK conceived and designed the evaluation and helped to draft the manuscript. CA participated in designing the evaluation and performed parts of the statistical analysis. BT re-evaluated the clinical data and revised the manuscript. UR evaluated and performed the statistical analysis and revised the manuscript. MO and IA collected the clinical data, interpreted them and revised the manuscript. MZ re-analyzed the clinical and statistical data and revised the manuscript. All authors read and approved the final manuscript.

**Table 1:** Diagnosis and time. Turnaround times and diagnoses according to DSM-IV, time intervals and mean with SD and median are also given.

Diagnosis	< 1 h	>1 h <3 h	>3 h	Mean(SD); median
Delirium, Dementia, Cognitive Disorders	11,5%	37,2%	51,3%	197 (SD 129); 182
Substance-Related Disorders	16,8%	55,6%	27,6%	139 (SD 102); 115
Schizophrenia and Other Psychotic Disorders	42,8%	47,6%	9,6%	89 (SD 78); 65
Mood Disorders	25,6%	57,8%	16,6%	118 (SD 94); 90
Anxiety Disorders, Adjustment Disorders	26,4%	52,2%	21,4%	120 (SD 90); 95
Personality Disorders	21,8%	53,6%	13,6%	98 (SD 73); 80

## References

[B1] Baker B Rochon J Lenth of stay, short stay units and psychiatric emergency admissions Can J Psychiatry 1989 34 39 42 2924247 10.1177/070674378903400110

[B2] Naumann S Miles JA Managing waiting patient's perceptions. The role of process control J Manag Med 2001 15 376 386 10.1108/EUM0000000006184 11765320

[B3] Summers M Happell B Patient satisfaction with psychiatric services provided by a Melbourne tertiary hospital emergency department J Psychiatr Ment Health Nurs 2003 10 351 357 10.1046/j.1365-2850.2003.00600.x 12755921

[B4] Kihlgren AL Nilsson M Skovdahl K Palmblad B Wimo A Older patients awaiting emergency department treatment Scand J Caring Sci 2004 18 169 176 10.1111/j.1471-6712.2004.00266.x 15147480

[B5] Walrath JM Tomallo-Bowman R Maguire JM Emergency Department: Improving Patient Satisfaction Nurs Econ 2004 22 71 74 15108475

[B6] Kissling W Seemann U Piwernetz K Quality management in psychiatry Int Clin Psychopharmacol 2001 16 suppl 3 S15 S24 10.1097/00004850-200104003-00003 11459328

[B7] Schnyder U Klaghofer R Leuthold A Buddeberg C Characteristics of psychiatric emergencies and the choice of intervention strategies Acta Psychiatr Scand 1999 99 179 187 10100912 10.1111/j.1600-0447.1999.tb00974.x

[B8] Southall AC Harris VV Patient ED turnaround times: a comparative review Am J Emerg Med 1999 17 151 153 10.1016/S0735-6757(99)90049-9 10102315

[B9] Chan L Reilly KM Salluzzo RF Variables That Affect Patient Turnaround Times in an Academic Emergency Department Am J Med Qual 1997 12 183 186 9385728 10.1177/0885713X9701200403

[B10] Lewin Group (for) the American Hospital Association) Emergency department overload: a growing crisis: The results of the American Hospital Association Survey of Emergency Department (ED) and Hospital Capacity 2002 Falls Church, VA: American Hospital Association

[B11] Trzeciak S Rivers EP Emergency department overcrowding in the United States: an emerging treat to patient safety and public health Emerg Med J 2003 20 402 405 10.1136/emj.20.5.402 12954674 PMC1726173

[B12] Hepp U Moergeli H Trier SN Milos G Schnyder U Attempted Suicide: factor Leading to Hospitalization Can J Psychiatry 2004 49 736 742 15633851 10.1177/070674370404901104

[B13] Donabedian A Criteria, norms and standards of quality: what do they mean? Am J Public Health 1981 71 409 412 7468883 10.2105/ajph.71.4.409 PMC1619670

[B14] Donabedian A The seven pillars of quality? Arch Pathol Lab Med 1990 114 1115 1118 2241519

